# Smart Manhole Cover with Tumbler Structure Based on Dual-Mode Triboelectric Nanogenerators

**DOI:** 10.3390/s26092590

**Published:** 2026-04-22

**Authors:** Bowen Cha, Jun Luo, Zilong Guo

**Affiliations:** 1 School of Mechatronic Engineering and Automation, Shanghai University, Shanghai 200444, China; 2 State Key Laboratory of Mechanical Transmission, College of Mechanical and Vehicle Engineering, Chongqing University, Chongqing 400044, China

**Keywords:** TENG, smart city, manhole cover alarm, water immersion, displacement

## Abstract

Aiming at the technical pain points of traditional manhole covers with low intelligence high cost and excessive power consumption, this study designs a TENG-based alarm device to enhance the safety and maintenance efficiency of urban infrastructure. The device integrates a water immersion sensor and a displacement sensor enabling real-time status monitoring through a unique TENG mechanism. The solid–liquid mode water immersion sensor detects seepage through the triboelectrification effect. Water droplets contact electrodes on the surface of FEP film and generate electric energy to trigger the detection circuit. The displacement sensor adopts the independent layer mode of TENG and combines with a mechanical tumbler mechanism to realize displacement detection. External force-induced manhole cover displacement drives internal balls to roll and rub against electrodes. Electric energy is then generated to activate the detection circuit. On the basis of the two sensors, an efficient and reliable intelligent alarm system is constructed. The system receives and analyzes displacement and water immersion-sensing signals in real time. It rapidly identifies potential safety hazards including displacement offset water accumulation and leakage. Signal analysis and early warning prompts are completed synchronously. This system provides accurate and real-time data support for public facility monitoring, pipe network operation and maintenance, and regional security in smart cities. It helps achieve early detection and early disposal of hidden dangers and improves the intelligent and refined level of smart city monitoring.

## 1. Introduction

As key nodes of urban underground infrastructure, manhole covers are widely distributed in public areas such as roads and sidewalks. They play a core role in protecting underground pipe networks and ensuring the safety of pedestrians and vehicles, with their operational stability directly related to urban public safety and residents’ quality of life. With the in-depth advancement of smart city construction, the limitations associated with the passive protection of traditional manhole covers have become increasingly prominent, creating an urgent demand for the development of intelligent systems featuring active sensing and intelligent early warning [1,2,3,4,5]. By integrating sensing and communication technologies, smart manhole covers can monitor in real time abnormal states such as manhole cover movement and water immersion, providing accurate operation and maintenance data for urban management departments. This effectively reduces safety accidents caused by manhole cover problems, improves the level of refined urban management, and renders them an important part of the intelligent transformation of smart city infrastructure [6,7,8,9].

In the scenario of manhole cover abnormal state detection, the core demand is to achieve efficient and reliable sensing of movement and water immersion, but existing detection technologies still suffer from many urgent limitations. Current commercial or research-based manhole cover detection schemes are mostly constructed based on traditional sensors, such as diffuse reflection infrared sensors, gravity sensors, capacitive water immersion sensors, etc., whose detection principle mainly relies on light occlusion, gravity changes, or capacitance changes to identify manhole cover displacement, tilt, and water immersion states [10,11,12,13,14]. However, these schemes generally face three core problems. First, energy supply relies on built-in batteries or external power sources, which not only requires regular battery replacement to increase operation and maintenance costs, but it also has difficulty adapting to long-term unattended outdoor scenarios. Second, the cost of core components and assembly is relatively high, restricting large-scale promotion and application. Therefore, developing a low-power, simple-structured and cost-controllable detection technology has become a key breakthrough in promoting the large-scale implementation of smart manhole covers [15,16,17].

Since its proposal in 2012, TENG has achieved efficient capture of low-frequency, weak mechanical energy in the environment and conversion into electrical energy based on the coupling effect of triboelectrification and electrostatic induction. With its unique advantages, it has shown broad application prospects in many fields and provided an innovative solution to solve the technical limitations of traditional sensors [18,19]. In the field of intelligent sensing, the self-powered characteristic of TENG enables it to complete signal sensing and output without relying on external power sources, and it has been successfully applied in scenarios such as human motion monitoring, environmental vibration monitoring, and fluid detection [20,21]. In the field of infrastructure monitoring, TENG has been utilized for railway track state monitoring, transmission line breeze vibration monitoring, building structure health monitoring, etc. Its high-sensitivity response to low-frequency mechanical signals can accurately capture minor abnormalities of infrastructure and provide reliable data for operation and maintenance early warning [22,23]. Meanwhile, manhole cover movement and water immersion impact are both classified in the category of low-frequency mechanical signals, so the technical characteristics of TENG can be perfectly adapted to this scenario [24,25,26,27]. The technical advantages and application experience of TENG provide a new technical path for the design of smart manhole cover sensing systems, which is expected to break through the bottlenecks of energy supply, integration difficulty, and cost of existing traditional schemes, promote the development of self-powered, low-cost, high-reliability, and wide-adaptability smart manhole covers, and provide core technical support for the intelligent upgrading of smart city infrastructure [28,29].

This study proposes a smart manhole cover based on TENG, integrating two functional modules: abnormal movement detection and water immersion detection. The core design is the integrated integration of dual-mode TENG sensors. The abnormal movement detection module employs a structure consisting of a tumbler-type hemispherical cavity and multiple balls, specifically placing 15 FEP balls with a diameter of 10 mm in the hemispherical cavity, and 4 fan-shaped aluminum (Al) electrodes attached to the inner wall of the cavity. When the manhole cover undergoes abnormal movements such as prying, displacement or overturning, the FEP balls roll along the inner wall of the cavity under the action of inertia and gravity, generating continuous contact–separation movement with the Al electrodes. Based on the coupling effect of triboelectrification and electrostatic induction, electrons are driven to migrate between the electrodes, forming alternating current signals. The water immersion detection module is constructed of FEP film and dual Al electrodes. When water droplets or water flow contact the electrodes on the surface of the FEP film, the solid–liquid triboelectrification effect induces charge transfer between the two electrodes, realizing self-powered sensing of water immersion status. Systematic performance tests were carried out based on a self-built simulated manhole cover experimental platform. The results show that the abnormal movement detection module equipped with 15 FEP balls with a 10 mm diameter has the optimal comprehensive performance: under the conditions of a driving frequency of 4 Hz and a flip angle of 45°, the peak output voltage and current can reach over 60 V and 200 nA, respectively, and the signals remain stable under different frequencies (2 Hz, 4 Hz, 6 Hz) and angles (15°, 45°, 75°). The water immersion detection module can generate stable electrical signals for water droplets of different falling heights (5 cm, 10 cm, 15 cm), and the output at a height of 5 cm is about 20 V and 10 μA. Cross-validation experiments of the dual modules reveal that the signal coupling interference between the abnormal movement detection module and the water immersion detection module is almost zero, and both can work independently and accurately. On this basis, this study constructs an intelligent manhole cover monitoring system integrated with solid–liquid TENG. Through experimental tests simulating the displacement and water immersion states of the manhole cover, the sensor system verifies the function of real-time signal monitoring. The smart manhole cover proposed in this study realizes high environmental adaptability, high sensitivity, and low-cost monitoring of manhole cover abnormal movement and water immersion status, breaks through the bottleneck of high operation and maintenance costs of traditional manhole covers, provides a new technical solution for the intelligent transformation of urban underground infrastructure, and has important practical application value for promoting the construction of smart cities.

## 2. Result and Discussion

### 2.1. Structural Design

**Figure 1:**
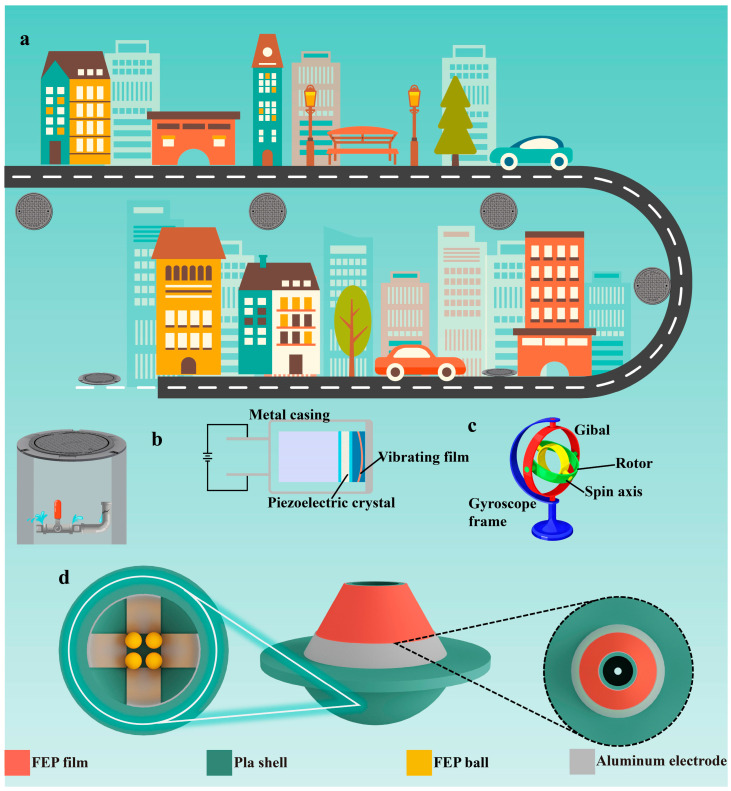
Application positioning and overall structure of the TENG-based smart manhole cover monitoring system. (**a**) Schematic diagram of the core application positioning of smart manhole covers in the smart city infrastructure system. (**b**) Principle of water immersion state detection for smart manhole covers. (**c**) Principle of abnormal movement state detection for smart manhole covers. (**d**) Schematic diagram of the overall structure of the TENG-based manhole cover alarm system.

Figure 1a intuitively presents the core application scenarios of smart manhole covers in smart city infrastructure. In the environment of high-density urban road networks, as terminal nodes of underground facilities such as drainage, power, and communication pipe networks, the operating status of manhole covers is directly related to urban public safety and operation as well as maintenance efficiency. The core value of smart manhole covers resides in the transformation of conventional passive protection manhole covers into intelligent terminals with active sensing capabilities. By real-time monitoring abnormal states and feeding them back to the urban management platform, it realizes accurate early warning of risks such as manhole cover theft, displacement, and water immersion, providing underlying data support for the refined management of smart cities. Based on the above application requirements, Figure 1b illustrates the principle of water immersion detection. The core design is based on an electrode conduction-triggered alarm. By placing a pair of spaced conductive electrodes inside the manhole cover, the water can act as a conductive medium to form a conductive path between the two electrodes once underground water accumulates and submerges the electrodes. Consequently, a circuit conduction signal is generated to identify the water immersion state. The advantage of this principle is its simple structure and direct response. Figure 1c shows a schematic diagram of the abnormal movement detection principle, in which an accelerometer serves as the core sensing component. The accelerometer is integrated inside the manhole cover. When the manhole cover experiences abnormal movements such as prying, displacement, or overturning, the internal inertial sensing unit detects variations in acceleration and outputs corresponding electrical signals, thereby realizing abnormal movement recognition via signal threshold judgment. The accelerometer-based detection method exhibits high response sensitivity, yet it presents obvious drawbacks in terms of anti-interference performance and battery life under long-term outdoor working conditions.

Aiming at the problems of poor stability, high price, and difficult fabrication existing in the current detection schemes, combined with the self-powered and high-sensitivity characteristics of TENG, a smart manhole cover integrated with dual-mode TENG sensors is designed. Its overall structure and core component design are shown in Figure 1d. The shell is integrally formed by 3D printing technology, and the material is a polylactic acid (PLA)-modified composite material. This material has excellent mechanical strength; installed under the manhole cover, it can effectively resist the impact of complex environments such as outdoor wind and rain erosion and vehicle rolling. The interior of the shell adopts a modular spatial layout design, and the installation areas of the solid–liquid mode TENG water immersion sensor, independent layer:layer mode TENG abnormal movement sensor, and signal processing module are planned through partitioned compartments. This not only ensures the working independence of each component but also facilitates later maintenance and debugging, and the physical diagram of its structure is depicted in Figure S1. The water immersion detection module is designed based on the solid–liquid TENG coupling effect, and its core sensitive unit is composed of a FEP film and Al electrodes. A FEP film is selected as the substrate, whose back is covered with an Al electrode as the back electrode; the front is selectively covered with a 2 cm wide strip-shaped Al electrode as the working electrode. Leads from the two electrodes are led out to the signal processing module through a waterproof connector. This design realizes water immersion sensing by changing the charge distribution between electrodes through liquid–solid contact, and the corresponding cross-sectional view and design dimensions are exhibited in Figure S2a,b. The abnormal movement detection module is designed based on the independent layer: layer mode TENG, adopting a spherical friction–electrode induction structure: the core component is a hemispherical cavity, on the inner wall of which fan-shaped Al electrodes are evenly attached and distributed at equal intervals along the circumferential direction of the cavity; FEP balls are placed inside the cavity, forming a friction-induction coupling pair with the electrodes on the inner wall of the cavity. When the manhole cover undergoes abnormal movement, the FEP balls roll along the inner wall of the cavity under the action of inertia, making contact–separation with Al electrodes at different positions, and induced electrical signals are generated through charge transfer between the electrodes, realizing self-powered detection of abnormal movement states. The corresponding cross-sectional view and design dimensions are displayed in Figure S2c. This device has a simple structure, high reliability, and low cost, and it has broad application prospects in the safety maintenance of urban infrastructure. Detailed design dimensions can be found in the Section 4.

### 2.2. Dynamic Characteristics of Displacement Module

**Figure 2:**
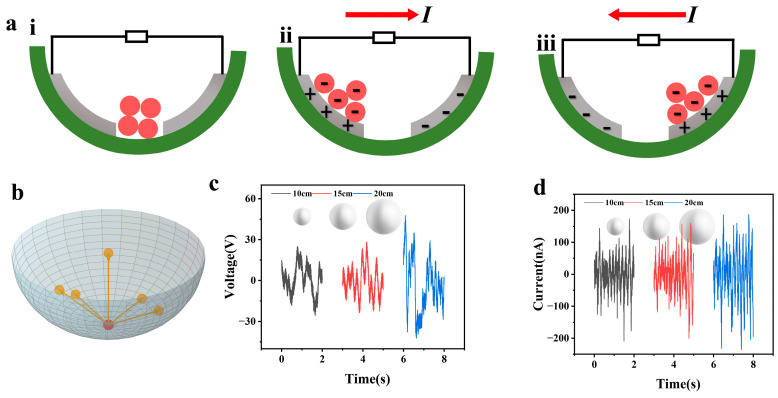
The working mechanism of the anomaly detection unit based on the independent layer mode TENG, and the influence of the ball’s degree of freedom and diameter. (**a**) Schematic diagram of the core working mechanism of the independent layer mode TENG anomaly detection unit. (**i**) Initial state; (**ii**) intermediate state; and (**iii**) final state of a cycle. (**b**) Schematic diagram of degree of freedom analysis of FEP balls in an inverted hemispherical cavity. (**c**) The open-circuit voltage and short-circuit current output characteristic curves of TENG anomaly detection units corresponding to FEP balls of different diameters. (**d**) The short-circuit current output characteristic curve of TENG anomaly detection unit corresponding to FEP balls of different diameters.

Figure 2a clearly illustrates the core working mechanism of the abnormal movement detection unit based on the independent layer mode TENG. According to the motion state of the FEP balls, the process of charge transfer and current generation can be divided into three key stages, namely the initial static state, the transitional rolling state from Electrode 1 to Electrode 2, and the transitional rolling state from Electrode 2 to Electrode 1.

In the initial static state (Figure 2a(i)), the FEP balls are stationary at the bottom of the hemispherical cavity, without contacting the Al electrodes (Electrode 1 and Electrode 2) on the inner wall of the cavity. At this time, no triboelectric charges are generated on the surfaces of the FEP balls and the Al electrodes, there is no potential difference between the two electrodes, no charge flows in the external circuit, and the current output is zero. When the manhole cover undergoes abnormal movement, the resultant force of inertia and gravity drives the FEP balls to break away from the static position, roll toward Electrode 1, and make contact with it. Due to the difference in triboelectric negativity between FEP and Al, a triboelectrification effect occurs at the contact interface between the two: electrons transfer from the surface of the Al electrode to the surface of the FEP balls, making the surface of the FEP balls negatively charged, and the surface of Electrode 1 in contact with them becomes positively charged due to the loss of electrons. As the FEP balls continue to roll on the surface of Electrode 1, the contact area gradually increases, the transferred charge accumulates synchronously, and the positive charge density on the surface of Electrode 1 continues to increase. When the FEP balls roll to the edge of Electrode 1 and start to transition to Electrode 2, electrons flow from Electrode 2 to Electrode 1 in the external circuit, generating a reverse current pulse (Figure 2a(ii)). When the FEP balls completely roll to the surface of Electrode 2 and continue to roll, the negative charges on the surface of the FEP balls undergo charge coupling with the surface of Electrode 2: positive charges are inductively generated on the surface of Electrode 2 under the action of the negative charge electric field, and the contact friction between the FEP balls and Electrode 2 further supplements the surface negative charges. When the FEP balls reset to the initial position under the tilting of the manhole cover and roll from the edge of Electrode 2 to Electrode 1 (reset transitional state), the direction of the electric field between the two electrodes reverses, and electrons flow from Electrode 1 to Electrode 2 in the external circuit, generating a reverse current pulse (Figure 2a(iii)). Afterwards, under the alternating action of inertia and gravity, the FEP balls roll back and forth between Electrode 1 and Electrode 2, and the above-mentioned charge transfer and current generation processes occur cyclically, forming a continuous alternating current output. It is worth noting that this design adopts rolling friction instead of traditional sliding friction, which not only reduces interface mechanical wear and improves the long-term durability of the detection unit, but also enhances the response sensitivity to micro-abnormal movements through the continuous change of contact area during the rolling process, ensuring the accurate capture of weak mechanical signals such as manhole cover prying and displacement.

Figure 2b shows the degree of freedom analysis of the FEP balls in the tumbler-type hemispherical cavity structure. Due to the hemispherical cavity design of this structure and the free rolling of the balls in the cavity, it has full-directional movement freedom and can roll around the center of the cavity in any horizontal direction without being restricted by specific directions. This full-degree-of-freedom movement characteristic can ensure that when the manhole cover undergoes abnormal movements such as prying, displacement, or overturning in any direction, the balls can form stable contact–separation movement with the Al electrodes on the inner wall of the cavity under the combined action of inertia and gravity, thereby ensuring that the abnormal movement detection module can accurately capture various abnormal states of the manhole cover and provide structural support for the subsequent stable electrical signal output.

To systematically explore the output performance of the designed TENG smart manhole cover abnormal movement detection unit, an experimental platform simulating manhole cover abnormal movement was built. By adjusting FEP ball diameter, with open-circuit voltage and short-circuit current as the core characterization indicators, the abnormal movement response performance test was carried out.

A comparative experiment was conducted with FEP ball diameter as the variable. Three different diameters of FEP balls were selected and respectively assembled into the hemispherical cavity of the detection unit. These diameters are 10 mm, 15 mm, and 20 mm. The other experimental conditions were kept consistent to eliminate the interference of irrelevant variables on the test results. Figure 2c and Figure 2d display the open-circuit voltage and short-circuit current output curves of the abnormal movement detection unit under different ball diameters, respectively. It can be seen from the test results that the output performance has a significant positive correlation with the FEP ball diameter: as the ball diameter increases from 10 mm to 20 mm, the peak open-circuit voltage increases accordingly, and the peak short-circuit current also increases. In addition, obvious signal fluctuation can be observed from the voltage output curve. This phenomenon is mainly due to the comprehensive coupling effect of inertia, gravity, and cavity wall friction on the rolling trajectory of the FEP balls in the cavity, which has a certain randomness, leading to slight differences in the contact position and contact timing between the balls and the electrodes, and ultimately causing fluctuations in the voltage signal. It is worth noting that this signal fluctuation characteristic precisely confirms the rationality and adaptability of the tumbler-type hemispherical cavity structure in this design. Specifically, the core advantages of the tumbler-type structure lie in its high sensitivity to external disturbances and effective motion guidance. The curved surface design of the hemispherical cavity provides a stable resting position for the FEP balls. In the absence of abnormal movement of the manhole cover, the balls can automatically return to this resting position under gravity, which maintains the detection unit in a low-power standby mode and prevents false triggering. Moreover, when the manhole cover suffers from abnormal movement in any direction, the inertial force drives the balls to roll along the curved cavity surface. The guidance effect of the curved surface ensures that the balls pass through the preset aluminum electrode area, thus inducing charge transfer and generating corresponding electrical signal outputs. The above-mentioned signal fluctuation is a direct reflection of the differential response of the balls to micro-abnormal movements under the guidance of the curved surface. Therefore, this signal fluctuation phenomenon not only does not affect the detection effectiveness, but also verifies the design logic that the tumbler-type structure can accurately capture micro-abnormal movements of the manhole cover and realize signal output through the differentiation of the ball rolling trajectory from the perspective of dynamic response, fully proving the rationality and superiority of this structure in the abnormal movement detection scenario.

### 2.3. Research on the Output Law of Displacement Module

**Figure 3:**
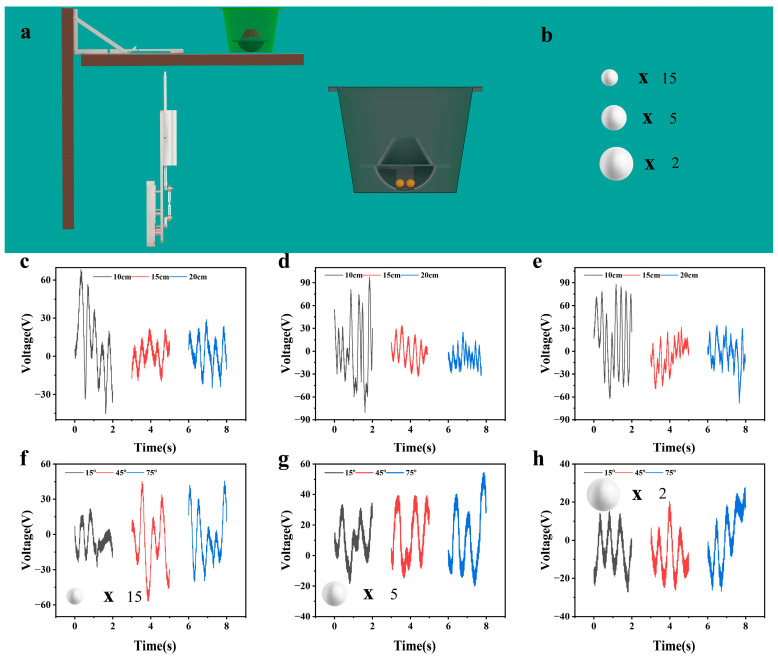
The influence of FEP ball diameter, motor drive frequency, and manhole cover flipping angle on the output performance of TENG anomaly detection unit. (**a**) Schematic diagram of building an integrated experimental platform for simulating abnormal movement of manhole covers. (**b**) Schematic diagram of different diameter combination FEP balls used in the experiment. (**c**–**e**) TENG open-circuit voltage output characteristic curves corresponding to different diameter combination FEP balls at different driving frequencies (2 Hz, 4 Hz, 6 Hz). (**f**–**h**) Output characteristic curves of combined FEP balls including 15 pieces of 10 mm diameter, 5 pieces of 15 mm diameter, and 2 pieces of 20 mm diameter at different manhole cover flipping angles under a driving frequency of 2 Hz.

To systematically explore the influence of FEP ball diameter, motor driving frequency, and manhole cover flip angle on the output performance of the abnormal movement detection unit, and clarify the optimal device parameter combination, an integrated experimental platform consisting of a simulated manhole cover, a driving system, and a testing system was built, and the experimental device is exhibited in Figure 3a. Among them, the designed abnormal movement detection unit was fixed at the center of the simulated manhole cover to ensure that the detection unit moved synchronously with the flat plate, which could truly reproduce the force state and movement law of the manhole cover during abnormal movement in practical application scenarios. The angle change and movement frequency of the manhole cover were realized by motor driving, and the motor was in contact with the center of the bottom of the simulated manhole cover through a rigid coupling. In this experiment, FEP ball diameter and motor driving frequency were taken as variables to carry out multiple sets of comparative tests. As shown in Figure 3b, three different specifications of FEP balls were selected in the experiment: 15 FEP balls with a diameter of 10 mm, 5 FEP balls with a diameter of 15 mm, and 2 FEP balls with a diameter of 20 mm. The driving frequency was set to three gradients of 2 Hz, 4 Hz, and 6 Hz (Figure S3a), covering the typical low-frequency disturbance range of daily abnormal movement of manhole covers. The manhole cover flip angle was fixed at 45°, which could effectively stimulate the rolling movement of FEP balls in the cavity and ensure the effectiveness of the test.

Figure 3c and Figure S3b show the open-circuit voltage and short-circuit current output curves of the three types of diameter balls under the driving frequency of 2 Hz. The test results present that the output performance of the 15 balls with a 10 mm diameter is significantly better than that of the other two specifications. The peak open-circuit voltage can reach up to about 60 V, and the peak short-circuit current can reach up to about 230 nA, while the maximum peak output voltages of the 5 balls with a 15 mm diameter and 2 balls with a 20 mm diameter are only about 22 V and 28 V, respectively, and the peak currents are also significantly lower than those of the 10 mm ball group. Figure 3d and Figure S3c display the output curves at a driving frequency of 4 Hz. Compared with the 2 Hz working condition, it can be seen that the output voltage and current of the three types of diameter balls all show different degrees of improvement. Among them, the peak voltage of the 10 mm ball group still maintains a stable level above 60 V, and the peak current remains around 200 nA. Figure 3e and Figure S3d illustrate the output curves at a driving frequency of 6 Hz. The data reveal that the peak output voltage and current of the three types of diameter balls are similar to those under the 4 Hz working condition, without obvious improvement, but the fluctuation amplitude of the voltage signal increases significantly. This signal characteristic precisely confirms the high sensitivity of the tumbler-type cavity structure. When the driving frequency reaches 6 Hz, the rolling speed of the balls accelerates, the randomness of the contact timing and contact position with the electrodes increases, leading to intensified dynamic fluctuations in the charge transfer process, and ultimately manifesting as unstable voltage signals. Meanwhile, the current signal does not exhibit obvious fluctuations, mainly because the current responds more directly to the contact frequency and is relatively less affected by fluctuations in the contact position. In addition, comparing the data of the 10 mm ball group under the three frequency conditions, it can be seen that its output voltage is above 60 V, showing excellent frequency adaptability. In terms of current output, the current of the 15 balls with a 10 mm diameter is always greater than that of the other two groups. Even under the high-frequency conditions of 4 Hz and 6 Hz, the current output of the 15 mm and 20 mm ball groups increases, but still does not exceed that of the 10 mm ball group, highlighting the advantage of the combined small-diameter balls in current output.

To explore the influence of manhole cover abnormal movement amplitude on the output performance of the detection unit, the motor driving frequency was fixed at 2 Hz to simulate manual conditions, and the manhole cover flip angle was set to three gradients of 15°, 45°, and 75°, corresponding to three typical abnormal states of manhole cover, including slight displacement, moderate prying, and severe flipping. Tests were carried out on the three types of diameters FEP ball groups, respectively. Figure 3f and Figure S4a show the output curves of the 15 balls with a 10 mm diameter at different angles. The results show that their output performance changes regularly with the increase in angle: the open-circuit voltage gradually increases from 20 V at 15° to 45 V at 75°, and the short-circuit current increases from 170 nA to 240 nA. Figure 3g and Figure S4b present the output data of the 5 balls with a 15 mm diameter at the same time. The peak voltage and current are both lower than those of the 10 mm ball group, and the increase amplitude with the increase in angle is not obvious. Figure 3h and Figure S4c reveal the output curves of the 2 20 mm balls, and the peak voltage and current are both lower than those of the 10 mm ball group. Comprehensive comparison of the output performance of the three types of diameter balls at the three angles shows that the combined 15 balls with a 10 mm diameter exhibits the optimal comprehensive performance with the highest peak output voltage and current. The core reason is that the combination of a large number of small-diameter balls can significantly increase the total contact area with the electrodes and improve the total charge transfer of triboelectrification. However, due to the small number of large-diameter balls, the contact area with the electrodes is limited, and they are prone to large-scale shaking under the influence of angle changes during rolling, resulting in poor signal stability. Continuing to reduce the diameter of the ball under the same total volume will help improve electrical output, thereby enhancing stability and sensitivity. However, this design also leads to relatively high costs for the entire system. Therefore, selecting the appropriate ball size requires a comprehensive consideration of output performance and economic benefits.

### 2.4. Dynamic Characteristics of Water Immersion Module

**Figure 4:**
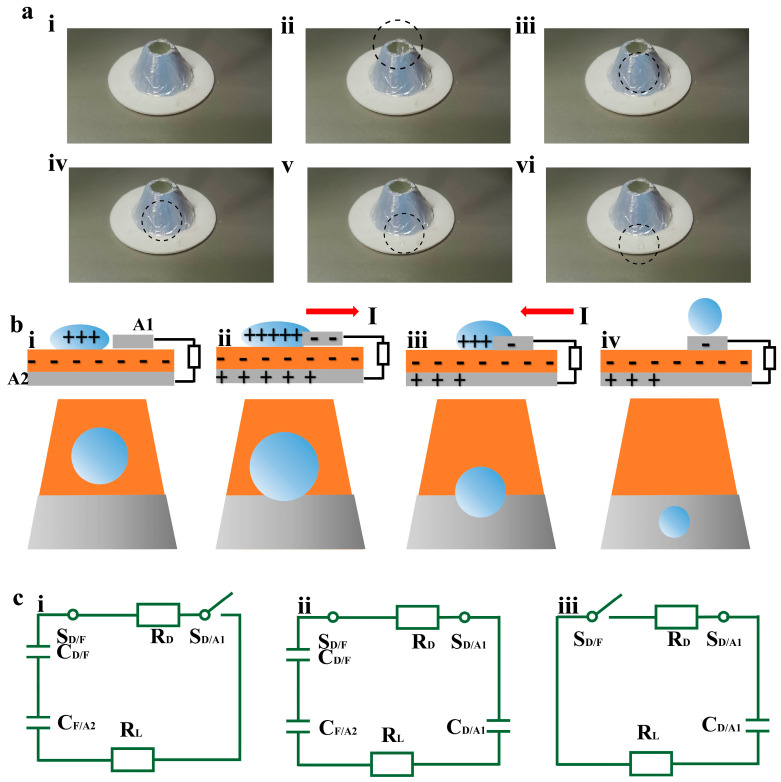
Verification of TENG universality. (**a**) The motion trajectory and dynamic modeling of water droplets in DTENG. (**i**) Initial stationary stage. (**ii**) Water droplet free falling stage. (**iii**) Water droplet expanding state on FEP film. (**iv**) Water droplet contracting state on FEP film. (**v**) Water droplet sliding and detachment stage. (**vi**) Water droplet shape restoration stage. (**b**) Working mechanism of TENG. (**i**) The state where water droplets do not come into contact with the aluminum electrode on the FEP film. (**ii**) The state where the water droplet contacts the aluminum electrode in an expanded state. (**iii**) The state where the water droplet contacts the aluminum electrode in a contracted state. (**iv**) The State of water droplet separation from FEP film surface. (**c**) Establishment of DTENG equivalent circuit. (**i**) Circuit shutdown status. (**ii**) Circuit conductivity status. (**iii**) Circuit cutoff state.

When a single water droplet freely falls from a height of 15 cm and impacts the cylindrical surface coated with FEP film, its dynamic behavior is significantly different from that on a flat surface. The core difference arises from the geometric confinement effect of the cylindrical surface. The whole process can be divided into four stages: interface impact, spreading-slip, retraction, and retention. The interfacial properties and motion laws of each stage are as follows: As shown in Figure 4a(i,ii), when the water droplet impacts the FEP-coated cylindrical surface with the kinetic energy of free fall, the initial contact mode is point contact. At the moment of contact, the bottom of the droplet undergoes rapid flattening deformation under the supporting force and impact pressure from the curved surface, and it can spread along two dimensions. The axial direction is parallel to the cylinder axis and the circumferential direction is around the cylinder surface. As shown in Figure 4a(iii), after the impact inertia drives the droplet into the spreading stage, the geometric confinement of the cylindrical surface endows the spreading with directional characteristics. Under the combined action of inertia, surface tension, and interfacial adhesion, the spreading direction is distinctly differentiated: Axial spreading is hardly restricted by curvature, allowing the droplet to extend rapidly along the cylinder axis and form a long liquid film; circumferential spreading is suppressed by the curvature of the cylinder, preventing the droplet from spreading widely around the surface, so the spreading width remains limited, and the liquid film thickness gradually decreases from the contact point to both axial ends. After surface tension becomes the dominant force, the droplet enters the retraction stage (Figure 4a(iv)). During retraction, the elongated liquid film gradually converges toward the center, with increasing thickness, and the originally dispersed liquid gathers near the initial contact point. Viscous dissipation at the interface causes partial kinetic energy loss, making the retraction velocity lower than the spreading velocity. As shown in Figure 4a(v,vi), the final state of the droplet impacting the cylindrical surface is determined by the initial kinetic energy, interfacial adhesion, and cylinder curvature. Under the action of a certain inclination angle of the flat plate, the droplet slides downward. The detailed process can be seen in Video S1.

When a water droplet falls freely from a height of 15 cm and impacts the cylindrical surface coated with FEP film, dynamic charge separation and electron transfer occur in the system based on the coupling of triboelectric effect and electrostatic induction, eventually forming a detectable electrical signal output. The core mechanism of this process originates from the strong electronegativity of FEP and the morphological evolution of the droplet under cylindrical confinement. As a typical strongly electronegative polymer, FEP surface exhibits a significant electron-accepting tendency during solid–liquid contact. In this study, a core sensing structure of “FEP film-dual Al electrodes” is constructed by fully covering an Al electrode (denoted as A2) on the back of the FEP film and arranging another Al electrode (denoted as A1) on the front side. The entire charge transfer and electron migration process can be divided into the following key stages combined with Figure 4b,c. After the droplet impacts the FEP film surface, driven by impact inertia and the guidance of the cylindrical surface, the droplet does not spread uniformly in all directions as on a flat surface, but extends directionally along the axial direction of the cylinder to form an elongated liquid film. This expansion rapidly increases the contact area between the droplet and the FEP film: more electrons transfer from the droplet to the FEP surface, greatly increasing the negative charge density on the FEP surface, and the total positive charge carried by the droplet increases synchronously (Figure 4b(i),c(i)). As the liquid film expands directionally, when the droplet touches the front Al electrode A1 of FEP, the equivalent electric field inside the system begins to drive electron migration: due to the electrostatic induction of the positive charges carried by the droplet on A1, electrons flow directionally from A2 to A1 through the external circuit, forming a transient current output. At this time, the equivalent circuit changes from open to closed. The electric double layer formed at the interface consists of a negatively charged FEP surface and positively charged droplets. This structure can be equivalent to a capacitor $C_{D/F}$ (D is the water droplet, F is the FEP film), and the positive charges stored in this capacitor begin to release through the circuit. Meanwhile, equivalent capacitors $C_{D/A1}$, $C_{F/A2}$ are formed between A1 and the droplet and between A2 and the FEP film, respectively, which are charged synchronously under the electric field, completing the initial charge storage and distribution.

When the droplet expands to its maximum extent, the contact area with the FEP film reaches a peak, the charge distribution at the solid–liquid interface tends to be stable, and the electron migration from A2 to A1 continues, macroscopically showing a continuous increase in the output grounding current (Figure 4b(ii),c(ii)). Droplet contraction–detachment stage: After the expansion inertia of the droplet decays, it contracts directionally along the cylindrical surface dominated by surface tension. The liquid film area gradually decreases, and the contact region between the droplet and the FEP film shrinks step by step. The voltage quickly drops to zero, and electrons flow rapidly from A1 to A2 (Figure 4b(iii),c(iii)). When the contracted droplet rests on the Al electrode A1, $C_{F/A2}$ and $C_{D/F}$ disappear accordingly, and the switch $S_{D/F}$ is open. As the droplet further contracts and finally detaches from the FEP film surface, the charge separation state at the solid–liquid interface disappears, the equivalent circuit is disconnected again, the electron migration process terminates, and the current output returns to zero (Figure 4b(iv),c(iii)).

According to Kirchhoff’s laws of voltage and current, the entire process of continuous contact between water droplets, FEP film, and electrodes can be described by the following differential equation. The detailed derivation process can be found in the supporting data (Note S1, Supporting Information)

(1) $$\frac{Q_{0}-q\left(t\right)}{C_{D/F}\left(t\right)}-\frac{dq\left(t\right)}{dt}R_{D}-\frac{q\left(t\right)}{C_{D/A1}\left(t\right)}-U_{R_{L}}\left(t\right)-\frac{q\left(t\right)}{C_{F/A2}\left(t\right)}=0$$

(2) $$\frac{dq\left(t\right)}{dt}-\frac{U_{R_{L}}\left(t\right)}{R_{L}}=0$$

(3) $$C_{D/F}\left(t\right)=\frac{\varepsilon_{D}S_{FEP}\left(t\right)}{d_{EDL}}$$

(4) $$C_{D/A1}\left(t\right)=\frac{\varepsilon_{D}S_{A1}\left(t\right)}{d_{EDL}}$$

(5) $$C_{F/A2}\left(t\right)=\frac{\varepsilon_{F}S_{FEP}\left(t\right)}{d_{FEP}}$$

where $Q_{0}$ represents the accumulated positive charge of $C_{D/F}$ when the droplet contacts Al1, q(t) represents the amount of charge transferred in the circuit, $U_{R_{L}}$ represents the voltage across the load, and $\varepsilon_{D}$ and $\varepsilon_{F}$ represent the capacitance of the droplet and FEP, respectively. In addition, $S_{FEP}\left(t\right)$ and $S_{Al}\left(t\right)$ represent the contact area between the droplet and the surfaces of FEP and Al1. $d_{EDL}$ represents the thickness of the double layer, and $d_{FEP}$ represents the thickness of FEP.

### 2.5. Research on the Output Law of Water Immersion Module

**Figure 5:**
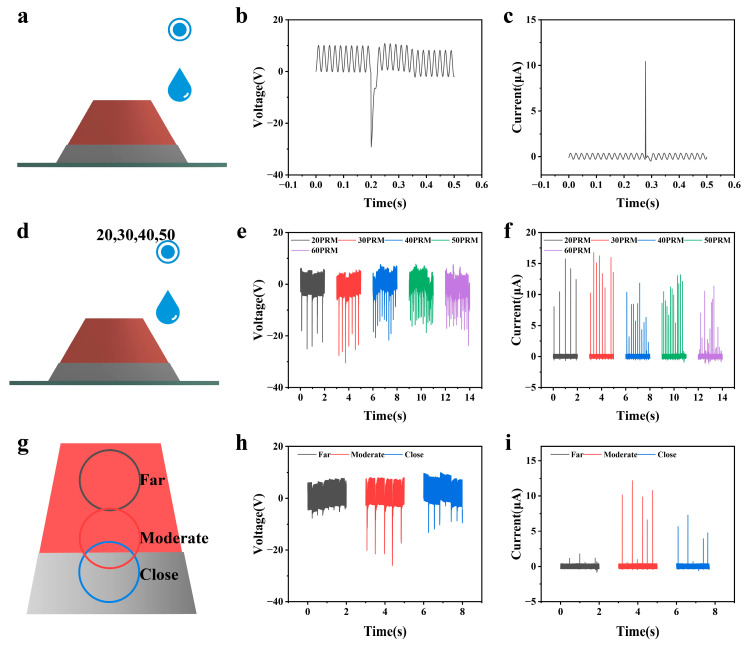
Verification of the influence of water droplet impact behavior on the output of TENG electricity. (**a**) Schematic diagram of water droplet impacting FEP film. (**b**,**c**) The voltage and current signal characteristics of a droplet based on TENG. (**d**) Schematic diagram of water droplet velocity as a variable. (**e**,**f**) The voltage and current output at different water droplet velocities. (**g**) Schematic diagram of the landing point of water droplets as a variable. (**h**,**i**) The voltage and current output at different landing points of water droplets.

Figure 5a–c completely and clearly reproduce the full-cycle dynamic process of a single water droplet falling freely from a height of 25 cm, impacting the surface of the cylinder coated with FEP film, and finally detaching from the FEP substrate. From the perspective of signal characteristics, the electrical signal undergoes a complete fluctuation cycle: zero baseline, peak jump, reverse drop, and return to zero. The stage where the signal jumps from the baseline corresponds to the process where the water droplet makes contact with the FEP surface and expands directionally along the axial direction of the cylinder. At this time, the increase in the solid–liquid contact area enhances the triboelectrification effect, and the increase in the amount of charge separation promotes the rise of open-circuit voltage and the formation of positive pulses of a short-circuit current. The stage where the signal gradually drops after reaching the peak corresponds to the process where the water droplet starts to shrink directionally after expanding to the maximum range. The decrease in contact area leads to the reconstruction of interface charge distribution, and the open-circuit voltage attenuates synchronously; the final return of the signal to zero indicates that the water droplet is completely detached from the FEP substrate, the triboelectrification and electrostatic induction processes at the solid–liquid interface terminate, and the system returns to a charge-neutral state.

The experimental scheme and test equipment are posted in Figure 5d and Figure S5. We further studied the influence of water droplet speed. Figure 5e,f present that when the flow rate increases from 20 RPM to 30 RPM, both voltage and current display a slight upward trend. This is mainly attributed to the increase in water droplet frequency, which leads to an increase in the number of contact–separation cycles per unit time, thereby improving the efficiency of charge accumulation and release. However, when the flow rate continues to increase to 50 RPM, the output voltage and current actually decrease. The reason for this abnormal phenomenon is that when the frequency of water droplets is too high, the next water droplet arrives at the surface before the previous water droplet completes the expansion, contraction and retraction process, resulting in overlap and interference of the expansion and contraction processes of water droplets. The interface contact behavior is no longer complete, thereby weakening the triboelectrification effect and charge induction effect.

When further exploring the energy conversion characteristics of the droplet–FEP film–Al electrode composite structure, the sizes of the FEP film and Al electrode were kept unchanged to systematically study the output behavior of the droplet when it contacts the electrode during the expansion and contraction process. The experimental results show that there are significant differences between the three typical cases. As depicted in Figure 5g, when water droplets fall on the FEP film, they only contact the front Al electrode after completing the full expansion and contraction process (this state is defined as “far”). As exhibited in Figure 5h,i, the output voltage in this case is only about 5 V, and the current is also very weak. This phenomenon may be due to the extremely short charge storage time generated during the diffusion stage of the droplet contact with the FEP interface. When the droplet finally contacts the Al electrode, the interface charge has been greatly reduced, leading to a significant weakening of the charge induction effect and near-disappearance of the output signal. Secondly, when the water droplet contacts the aluminum electrode during the expansion process, this state is defined as moderate. The output voltage reaches about 25 V and the current is about 12 μA, which is much higher than other modes. At this time, the droplet is still in the high-speed distribution stage, the contact area increases rapidly, the interface charge concentration is high, and the transient potential difference is large, which enables the most sufficient electron transfer between the rear electrode and the front electrode, thereby achieving the maximum energy conversion efficiency. The third case is that the water droplet directly falls on the FEP film and the front Al electrode (this state is defined as “close”), with an output voltage of about 10 V and a current of about 7 μA. Compared with the second mode, the interface charge induction effect is partially weakened, resulting in an output amplitude lower than that of the contact during the expansion process.

### 2.6. Universal Demonstration of Water Immersion Module

**Figure 6:**
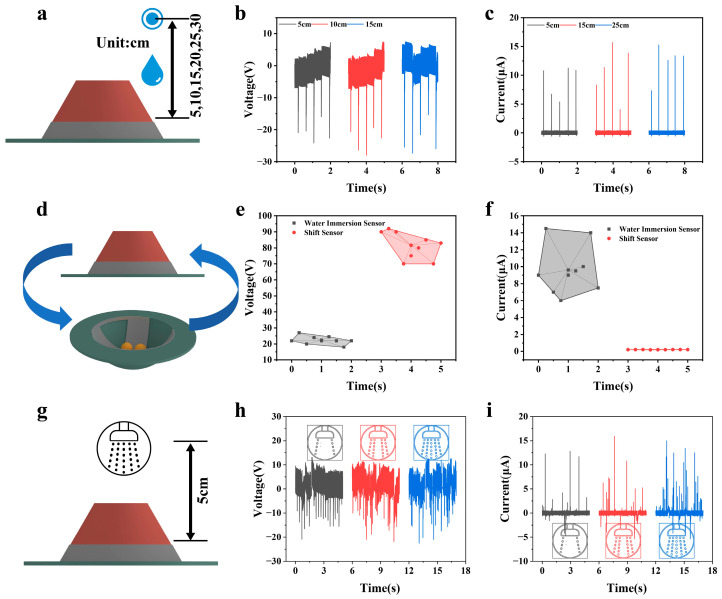
The impact of droplet drop height, dual-module compatibility, and complex water immersion scenarios on TENG output performance. (**a**) Schematic diagram of the test scenario for the influence of water droplet falling height on TENG output performance. (**b**,**c**) TENG open-circuit voltage and short-circuit current output characteristic curves corresponding to different water droplet falling heights. (**d**) Schematic diagram of the cross-validation experimental scenario for the collaborative work of anomaly detection and water immersion detection dual modules. (**e**,**f**) Output of open-circuit voltage and short-circuit current for dual-module collaborative operation. (**g**) Schematic diagram of TENG output performance testing scenario for complex water immersion scenarios. (**h**,**i**) The output characteristic curves of TENG open-circuit voltage and short-circuit current under different water volume gradients.

It provides stable experimental conditions for systematically exploring the correlation between water droplet falling height and electrical output performance. Based on this, we carried out targeted variable experiments: on the premise of keeping core parameters consistent, such as constant front electrode width and unchanged FEP film material and size, we systematically studied the output laws of the device’s open-circuit voltage and short-circuit current when the water droplet falling heights were 5 cm, 10 cm, and 15 cm, respectively. The test scenario is depicted in Figure 6a. The test results in Figure 6b,c clearly indicate that there is a significant positive correlation between the water droplet falling height and the electrical output performance of the device: when the falling height gradually increases from 5 cm to 15 cm, the device’s output voltage continuously rises from about 20 V to 25 V, and the output current significantly increases from about 10 μA to 15 μA. The core mechanism of this variation law can be explained from the perspectives of energy transfer and interface interaction mechanism: the increase in water droplet falling height directly converts its gravitational potential energy into greater kinetic energy before impacting the FEP film. When high-kinetic energy water droplets impact the FEP surface, they generate stronger impact pressure and more intense interface interaction. Greater impact kinetic energy can drive water droplets to spread along the FEP surface faster and more fully, significantly enhancing the contact–separation dynamic characteristics between water droplets and the FEP film, and greatly expanding the transient contact area of the solid–liquid interface. The increase in the amount of charge transfer directly promotes the rise in the induced potential difference between the electrodes, thereby realizing the improvement of the open-circuit voltage; at the same time, more accumulated charges form directional migration through the external circuit, which ultimately manifests as the synchronous enhancement of the short-circuit current. It is worth noting that even under the test condition of the lowest falling height of 5 cm, the output voltage of the device can still reach 20 V and the output current is about 10 μA, which fully meets the energy demand for the conduction of the subsequent driving circuit.

To verify the compatibility between the abnormal movement detection module and the water immersion detection module in the designed smart manhole cover, that is, to clarify whether there is signal coupling interference when the two work, a cross-validation experiment of the dual-module collaborative work was carried out, and the scenario is the drawing in Figure 6d. The core idea of the experimental design is as follows: while the water immersion detection module normally conducts the water droplet impact test, drive the FEP balls of the abnormal movement detection module to roll along the Al electrodes in its hemispherical cavity to simulate the abnormal movement state of the manhole cover, and record the electrical output of the water immersion sensor; when the FEP balls of the abnormal movement detection module roll along the Al electrodes in its hemispherical cavity, record the electrical output of the displacement sensor with different degrees of water dripping. The focus is on observing whether the open-circuit voltage and short-circuit current signals of the water immersion sensor have abnormal changes due to the work of the abnormal movement module, so as to determine the degree of coupling interference between the two. The test results in Figure 6e,f clearly show that during the collaborative work of the FEP balls of the abnormal movement detection module rolling along the electrodes, there is no significant abnormality in the electrical output signal of the water immersion sensor. Similarly, when water droplets fall, the output of the displacement sensor does not decrease. This experimental result fully proves that the signal coupling effect between the abnormal movement detection module and the water immersion detection module is almost zero. The core reason is that the working mechanisms and energy conversion paths of the two modules have clear independence. The water immersion sensor is based on the solid–liquid triboelectrification effect, and its electrical signal originates from the charge separation and transfer at the interface between water droplets and the FEP–Al electrode, with the energy carrier being the impact kinetic energy of water droplets. Meanwhile, the abnormal movement sensor is based on the solid–solid contact–separation triboelectrification effect, and the signal originates from the rolling contact–separation between FEP balls and Al electrodes, with the energy carrier being the rolling kinetic energy of the balls. The energy sources and charge generation mechanisms of the two are completely independent, and there is no cross-energy transfer.

To systematically verify the response characteristics of the designed device in actual complex water immersion scenarios, an experimental platform with multi-water volume gradients simulated by a shower head was built for the progressive water spray conditions with different intensities of water accumulation in the tumbler structure at the bottom of the manhole cover. The test scenario is displayed in Figure 6g. The experimental design idea is as follows: by adjusting the water outlet gear of the shower head (corresponding to different water volumes), simulate the progressive water immersion scenarios from a small amount of dripping water, medium water flow to a large amount of spray; fix the shower head at a specific height of 5 cm directly above the device to ensure that the water flow acts vertically on the FEP–Al electrode composite sensing surface; during the experiment, keep irrelevant variables such as the vertical distance between the shower head and the device constant, take only the water volume as the sole variable, and collect the open-circuit voltage and short-circuit current signals of the device in real time through an electrometer and a source meter to explore the influence law of water volume changes on the characteristics of electrical output signals. The test results presented in Figure 6h,i demonstrate a clear correlation between the stability of the device’s electrical output and the water volume. As the water volume from the shower head gradually increases, the voltage and current pulse signals, which are regular and distinguishable under low water volume conditions, become increasingly disorganized. This degradation is characterized by an unstable signal baseline, greater dispersion in pulse peak values, irregular pulse occurrence, and significant superposition interference from reverse signals. This experimental result intuitively reflects the response difference of the device under different water immersion intensities, providing experimental basis for the subsequent accurate identification in complex water immersion scenarios.

### 2.7. Application of Smart Manhole Covers

**Figure 7:**
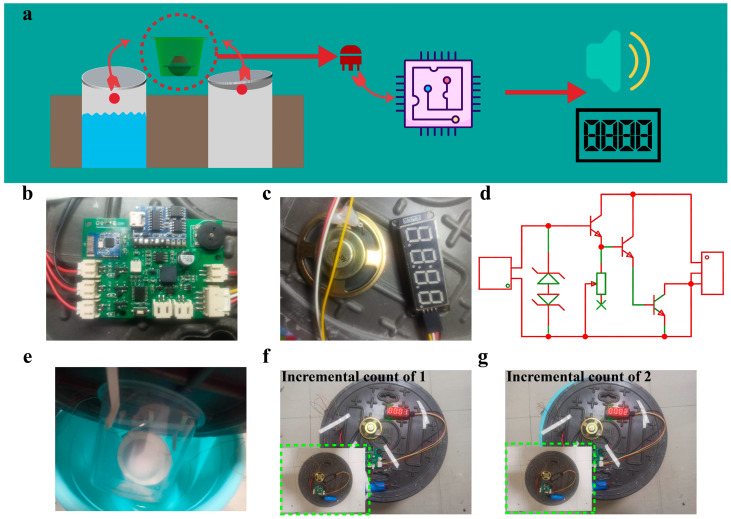
Application and related components of the smart manhole covers. (**a**) System information conversion process. (**b**) Core control system of the smart manhole covers. (**c**) Triode conversion circuit for TENG output signal conditioning. (**d**) System feedback components. (**e**) Real-time images demonstrating smart manhole covers. (**f**) Digital display of displacement status with incremental count of 1. (**g**) Digital display of water immersion status with incremental count of 2.

A small system can be integrated into the manhole cover alarm, which realizes the intelligent perception, identification, and management of a beautiful city through devices such as water immersion sensors and displacement sensors. Here, a central processing system is designed to complete the functions of the manhole cover alarm. The whole alarm system includes TENG modules, triode circuits, a single-chip microcontroller chip, a voice module, and a digital tube module. The operating principle between each module is displayed in Figure 7a. The core circuit and digital tube are depicted in Figure 7b and Figure 7c, respectively. When water droplets flow over the Al electrodes on the FEP surface, or when the FEP ball rolls on the electrodes, an electrical signal output is generated. However, excessive electrostatic voltage may damage the single-chip microcontroller system. Meanwhile, a triode is a semiconductor device with three electrodes, whose working principle is based on the current amplification effect of the semiconductor PN junction. When the output of the TENG is applied to the base of the triode, a current is generated between the base and the collector, turning on the collector and the emitter, thus achieving a switching function. As exhibited in Figure 7d, excessive voltage may cause air breakdown of the triode, so a TVS tube is connected in series. As shown in Figure 7e, the TENG module is connected to the alarm module with wires, and the connections between each module are checked to ensure a smooth circuit. The manhole cover alarm is then fixed on the manhole cover simulator to ensure close contact with the simulated manhole cover. Figure S6 illustrates the water flow path into the tumbler structure. A set of holes with a specific number are distributed at different positions on the top of the structure, ensuring that water droplets have a high probability of landing on the sensor surface.

As shown in Figure 7f, in the initial state, the working state of the alarm is observed and recorded. When the simulated manhole cover is lifted, the rolling ball moves inside and generates an electrical signal, and the working state of the alarm is observed and recorded. The corresponding number on the digital tube increases by 1. Meanwhile, it can be seen from Video S2 that the displacement sensor sends out an alarm signal, while the water immersion sensor shows no fluctuation, which proves the anti-interference performance of the manhole cover alarm. Subsequently, the simulated manhole cover is placed back on the test device, and the initial working state of the alarm is recorded (Figure 7g). Tap water is gradually poured into the water tank until it touches the nozzle at the bottom of the alarm. At the moment when water droplets slide down the FEP film, the signal of the alarm is recorded. Through the prompt function, the manhole cover alarm data before and after use can be compared, which can directly evaluate the effectiveness of the device. The corresponding number on the digital tube increases by 2. It can be seen from Video S3 that the TENG electrical output in the water immersion mode does not affect the fluctuation value of the displacement sensor, which remains stable. To verify the system response when the water immersion module is fully submerged in water, we conducted further experimental validation. Figure S7a shows the smart manhole cover water immersion sensing module completely immersed at the bottom of the water tank. However, as water droplets continuously fall onto the water surface and generate oscillations, the system shows no response (Figure S7b). This result indicates that the dynamic contact–separation process of water droplets on the FEP film is indispensable for triggering the circuit conduction; without such mechanical–electrical coupling dynamics, the circuit cannot be activated. This finding validates the data analysis and working mechanism of our water immersion sensor, confirming that the sensing relies on the triboelectric effect induced by droplet impact rather than simple static immersion.

The manhole cover alarm can perceive the state changes of the manhole cover in real time through its built-in displacement and water immersion sensor modules. Whether the manhole cover is moved illegally or an abnormal water overflow occurs, the alarm can quickly capture these changes and provide data support for timely response. Once an abnormal state of the manhole cover is detected, the alarm immediately starts the alarm procedure and sends out obvious alarm signals such as sound. These signals can not only remind nearby pedestrians and vehicles to pay attention to safety, but also quickly attract the attention of managers for timely response measures.

As shown in Table 1, existing works, research papers, and commercial products all rely on mature, off-the-shelf sensor components such as gyroscopes and accelerometers, combined with lithium sub-battery power supplies. While these studies have made incremental contributions in application-level algorithms and system deployment, no fundamental innovations have been made in the core sensor design itself. In contrast, our work directly targets the sensor-level innovation: we utilize the triboelectric effect to construct a self-powered sensing unit, eliminating the need for conventional inertial sensors and enabling energy harvesting from mechanical vibrations and water immersion.

Notably, to the best of our knowledge, this is the first reported TENG-based smart manhole cover alarm system, filling the gap in self-powered, low-cost sensing for municipal manhole cover monitoring. In terms of cost, our device achieves a single-unit cost of only about 5.8 dollar, which is 10–15 times lower than commercial products and existing academic prototypes. Although all comparative schemes rely on lithium sub-batteries with manual maintenance and limited waterproof performance, our TENG-based design enables self-powered sensing through mechanical energy harvesting, and the optimized sleep mode further reduces power consumption. While the current prototype still requires manual maintenance and has limited waterproofing, its self-powered characteristic, ultra-low cost, and novel sensor-level design make it a highly promising candidate for large-scale deployment in smart city infrastructure.

## 3. Conclusions

In this work, a TENG-based manhole cover alarm device is designed. By integrating a water immersion sensor and a displacement sensor, it realizes real-time and efficient monitoring of manhole cover status, with the merits of facile fabrication, low cost, and high stability. Benefiting from the solid–liquid and solid–solid sensing principles of TENG, the built-in TENG-based water immersion and displacement sensor modules require no external power supply, greatly facilitating its distributed deployment in smart cities. Notably, the TENG-based displacement sensor module and water immersion monitoring module are independent of each other without mutual interference. Furthermore, relying on the TENG-based manhole cover alarm, a full-process real-time sensing system is successfully constructed, integrating sensing detection, signal conditioning, data processing, and alarm notification. When the ambient environment changes dynamically, abnormal signals can be rapidly transmitted to the alarm module, thereby realizing real-time early warning for manhole cover displacement and water immersion hazards with high reliability and stability. In summary, the TENG-based manhole cover alarm device designed in this work exhibits favorable performance and effectiveness in practical applications. In the future, we will further optimize the performance of the device, explore its application potential in other fields, and provide more technical support and solutions for the safety maintenance of urban infrastructure.

## 4. Experimental Section

Fabrication of TENG-based dual-mode manhole cover structure: FEP films with strong electron-trapping capability were utilized. The FEP films are commercially available with a thickness of 0.1 mm, and no further surface modification was conducted prior to use. Commercially available FEP beads were also adopted, and no additional surface treatment was performed before utilization. In addition, Al electrodes with a thickness of 0.06 mm were employed, which are commercially available and require no extra surface modification prior to use. The entire housing was fabricated by 3D printing using PLA. Preparation of the water immersion module. The Al electrode was wrapped around the printed boss, and then FEP film was further coated onto the Al electrode. Finally, an Al electrode with a width of 1 cm was wound around the bottom of the boss. Preparation of the displacement module. Two Al electrodes with a width of 3 cm were directly attached inside a hemisphere with a diameter of 5 cm. Adjacent Al electrodes were connected together, the middle region was removed, and a certain number of FEP beads were placed inside.

Preparation of the intelligent system for smart manhole covers: A triode conversion circuit converts analog signals generated by the TENG into digital signals. These signals correspond to different working states including manhole cover displacement and water immersion. The digital signals are then detected and processed by a 51-series single-chip microcontroller (SCM). Subsequently, the SCM sends instructions to the alarm module according to the detected signals and controls the digital tube to display the count of abnormal events of the manhole cover.

Characterization and electrical measurements: The short-circuit current and open-circuit voltage were examined through a Keithley 6514 electrometer.

## Figures and Tables

**Table 1:** Comparison of typical smart manhole cover sensing systems.

Scheme Type	Working Principle	Power Supply Method	Approximately Cost (Dollar)	Maintenance Method
Mature IoT modules	Accelerometer; gyroscope	Lithium battery	57.8	Manual maintenance, not waterproof
Low power anomaly recognition type	Accelerometer; gyroscope	Lithium battery; sleep mode	28.9	Manual maintenance, not waterproof
Algorithm recognition	Electrode conduction module; Accelerometer; gyroscope	Lithium battery	86.5	Manual maintenance, not waterproof
Commercial products (www.taobao.com)	Electrode conduction module; Accelerometer; gyroscope	Lithium battery	43.4	Manual maintenance, not waterproof
TENG Sensors	Solid–liquid TENG; Solid–solid TENG	Lithium battery; sleep mode	5.8	Manual maintenance

## Data Availability

The data that support the findings of this study are available within the article and its Supplementary Material.

## Supplementary Materials

The following supporting information can be downloaded at: https://www.mdpi.com/article/10.3390/s26092590/s1. Supporting Note S1: The derivation process of circuit equations. Supporting Table S1: The descriptions of circuit elements used in this work. Figure S1: The corresponding photographs of the TENG device. Figure S2: Dual-mode TENG sensor core structure. Figure S3: TENG short-circuit current output characteristic curves corresponding to different diameter combination FEP balls at different driving frequencies. Figure S4: TENG short-circuit current output characteristic curve of different diameter combined FEP balls under different manhole cover flipping angles. Figure S5: The variable speed peristaltic pump in the experiment. Figure S6: Equipment schematic diagram in immersion mode. Figure S7: System response under fully water immersion condition. Video S1: The motion characteristics of liquid droplets on the FEP film coated on the upper part of a tumbler (MP4). Video S2: Manhole cover displacement sensing performance test (MP4). Video S3: Manhole cover water immersion sensing performance test (MP4).
