# Supplementary material for: Smart Manhole Cover with Tumbler Structure Based on Dual-Mode Triboelectric Nanogenerators
Source: Sensors (Basel). 2026 Apr 22;26(9):2590. doi: 10.3390/s26092590 (PMC13165642; doi:10.3390/s26092590)
Supplement: Supplementary file 1 [file sensors-26-02590-s001.zip › SI.pdf]

Supporting Information

Smart Manhole Cover with Tumbler Structure Based on Dual-Mode Triboelectric Nanogenerators

Bowen Cha,[a] *Jun Luo,*[b] *Zilong Guo*[*a]

[a] School of Mechatronic Engineering and Automation, Shanghai University, Shanghai 200444, China

[b] State Key Laboratory of Mechanical Transmission, College of Mechanical and Vehicle Engineering, Chongqing University, Chongqing 400044, China

* Correspondence: Email: Guozl@shu.edu.cn.

Supporting Videos

Supporting Video S1. The motion characteristics of liquid droplets on the FEP film coated on the upper part of a tumbler.

Supporting Video S2. Manhole cover displacement sensing performance test.

Supporting Video S3. Manhole cover water immersion sensing performance test.

Supporting Note S1. The derivation process of circuit equations.

According to Kirchhoff's voltage law, equation is as the following

$$U_{C_{D/F}}(t) - U_{R_D}(t) - U_{C_{D/AL1}}(t) - U_{R_L}(t) - U_{C_{F/AL2}}(t) = 0 \quad \text{(S1)}$$

$$C = \frac{Q}{U} \quad \text{(S2)}$$

$$\frac{Q_0 - q(t)}{C_{D/F}(t)} - \frac{d\mathrm{q}(t)}{dt}R_D - \frac{q(t)}{C_{D/AL1}(t)} - U_{R_L}(t) - \frac{q(t)}{C_{F/AL2}(t)} = 0 \quad \text{(S3)}$$

According to Kirchhoff's current law, equation is as the following

$$i(t) = i_{R_L}(t) \quad \text{(S4)}$$

$$\frac{dq(t)}{dt} - \frac{U_{R_L}(t)}{R_L} = 0 \quad \text{(S5)}$$

The initial conditions are

$$q(t = 0) = 0 \quad \text{(S6)}$$

$$U_R(t = 0) = 0 \quad \text{(S7)}$$

Supporting Table S1. The descriptions of circuit elements used in this work

Circuit Elements	Descriptions
$C_{D/F}$	Electric double layer capacitor formed between droplet and FEP surface
$C_{D/A1}$	Electric double layer capacitor formed between droplet and A_l 1surface
$C_{F/A2}$	Capacitor formed between FEP surface and Al_2
$S_{D/F}$	The switch indicating whether the droplet contacts the FEP surface
$S_{D/A1}$	The switch indicating whether the droplet contacts the Al 1surface
R_D	Resistance of the droplet
R_L	Resistance of the load

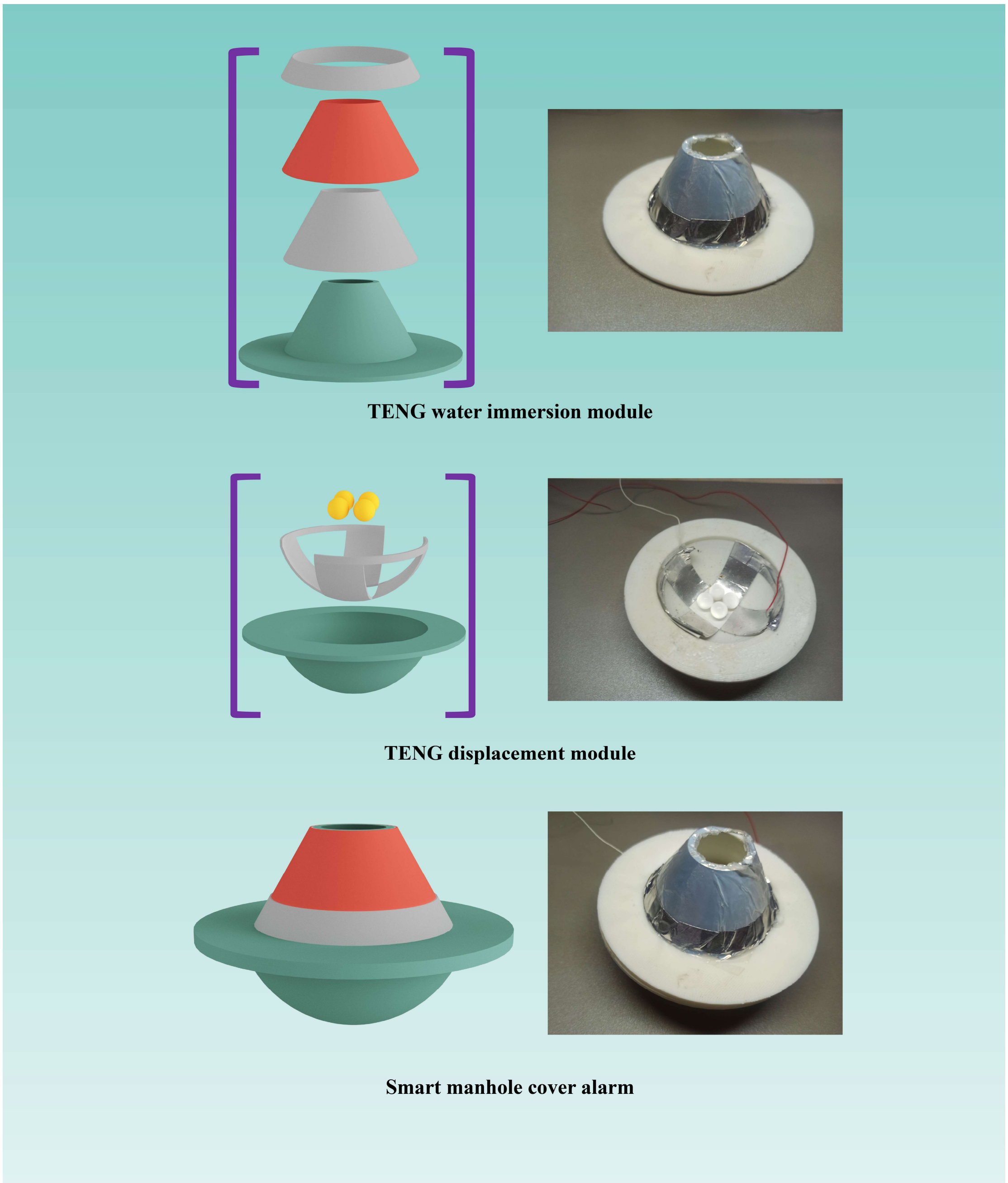

Figure S1. The corresponding photographs of the TENG device.

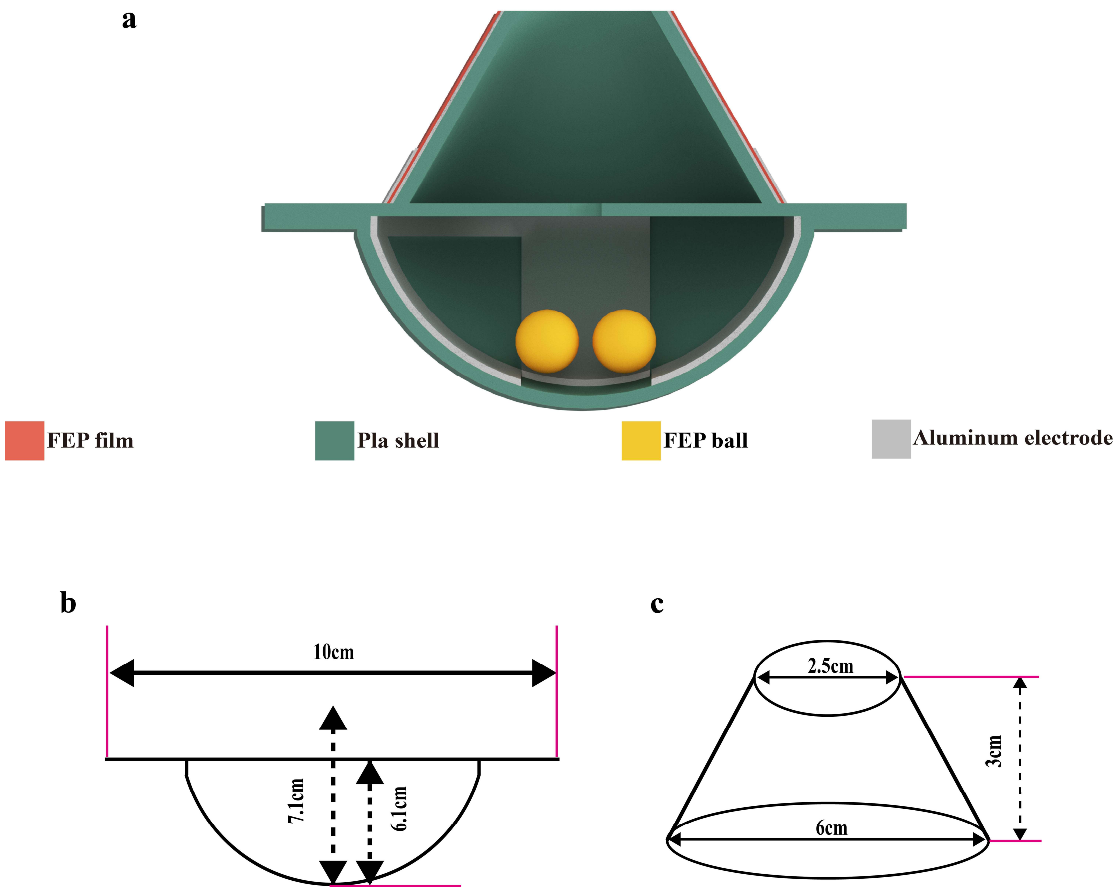

Figure S2. Dual mode TENG sensor core structure (a) Cross section diagram of the core structure of dual-mode TENG sensor. (b) Design details of the anomaly detection module. (c) Design details of the core sensitive unit of the water immersion detection module.

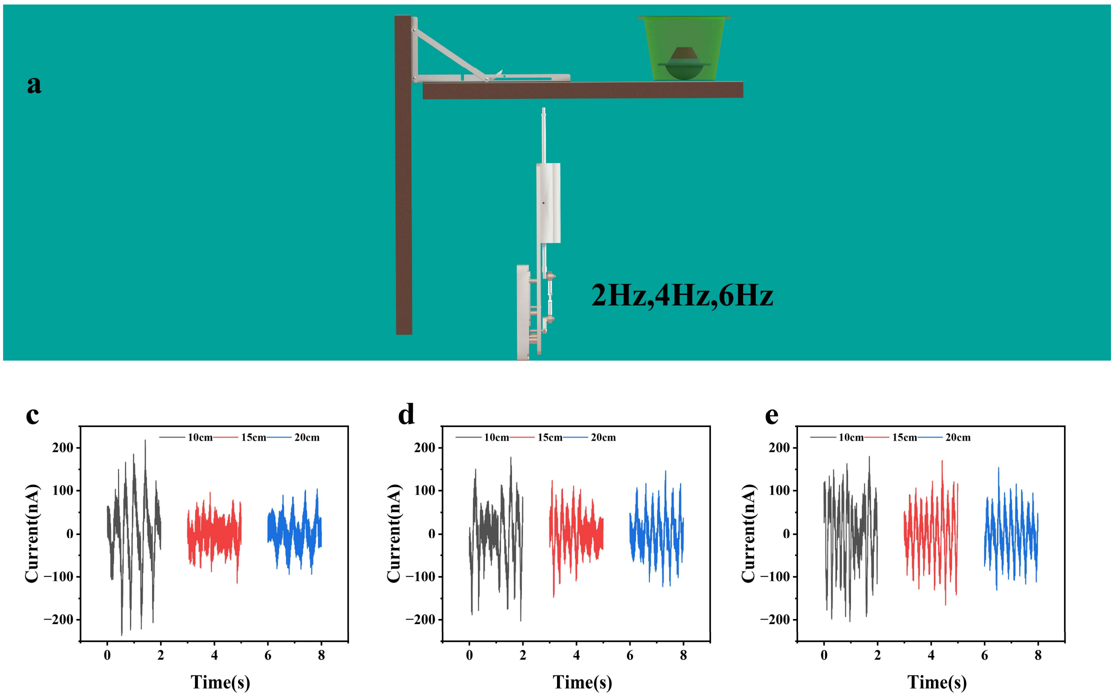

Figure S3. TENG short-circuit current output characteristic curves corresponding to different diameter combination FEP balls at different driving frequencies. (a)Schematic diagram of the frequency gradient setting for the motor drive used in Experiment. (b, c, d) TENG open circuit voltage output characteristic curves corresponding to different diameter combination FEP balls at different driving frequencies (2 Hz, 4 Hz, 6 Hz)

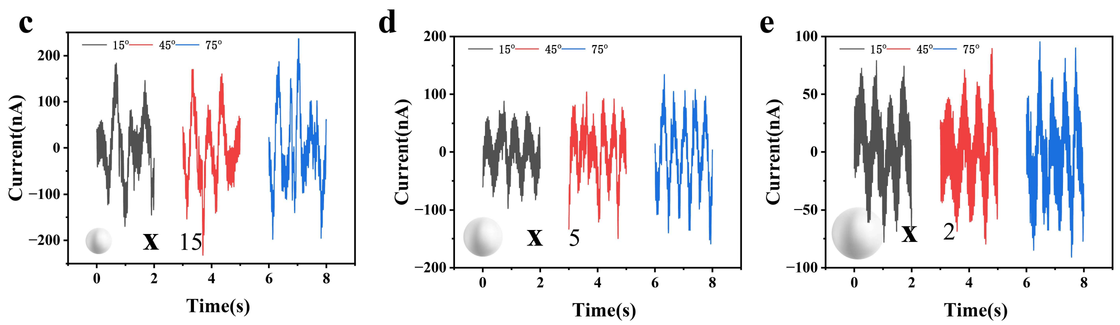

Figure S4. TENG short-circuit current output characteristic curve of different diameter combined FEP balls under different manhole cover flipping angles. (a, b, c) Output characteristic curves of 15 10 mm, 5 15 mm, and 2 20 mm combined FEP balls at different manhole cover flipping angles when driven at a frequency of 2 Hz.

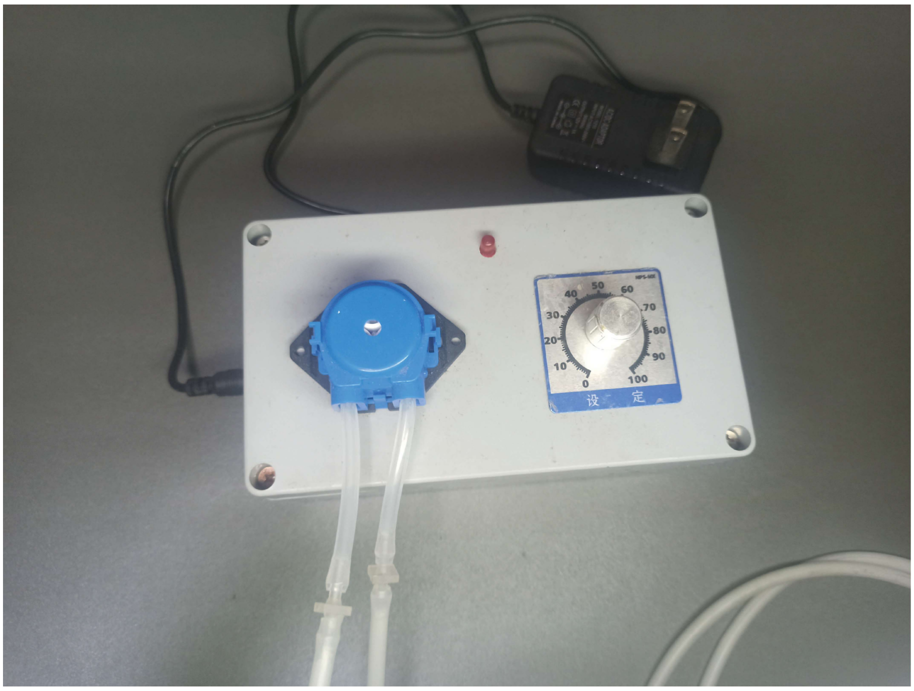

Figure S5. Variable speed peristaltic pump in the experiment.

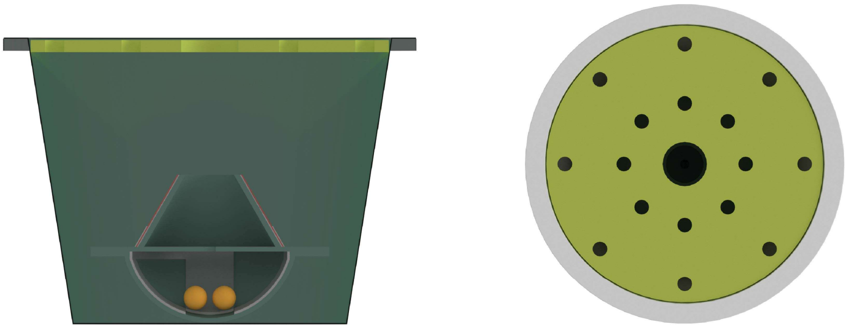

Figure S6. Equipment schematic diagram in immersion mode

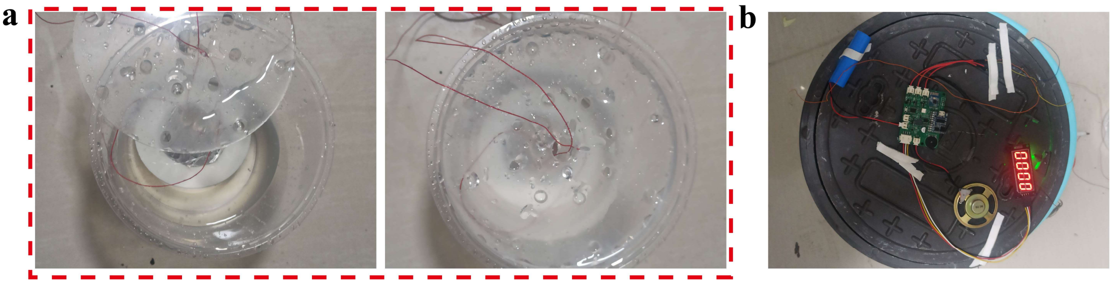

Figure S7. System response under fully water immersion condition. (a) Schematic of TENG module fully submerged in water. (b) System feedback signal of the monitoring system.
